# A 3D Wrist Pulse Signal Acquisition System for Width Information of Pulse Wave

**DOI:** 10.3390/s20010011

**Published:** 2019-12-18

**Authors:** Chuanglu Chen, Zhiqiang Li, Yitao Zhang, Shaolong Zhang, Jiena Hou, Haiying Zhang

**Affiliations:** 1 Institute of Microelectronics of Chinese Academy of Sciences, Beijing 100029, China; chenchuanglu@ime.ac.cn (C.C.); zhangyitao@ime.ac.cn (Y.Z.); zhangshaolong@ime.ac.cn (S.Z.); houjiena@ime.ac.cn (J.H.); zhanghaiying@ime.ac.cn (H.Z.); 2 University of Chinese Academy of Sciences, Beijing 100049, China; 3 Beijing Key Laboratory for Next Generation RF Communication Chip Technology, Beijing 100029, China

**Keywords:** micro-electro-mechanical system (MEMS), traditional Chinese medicine (TCM), flexible sensor array, radial artery, width information, wrist pulse signal

## Abstract

During pulse signal collection, width information of pulse waves is essential for the diagnosis of disease. However, currently used measuring instruments can only detect the amplitude while can’t acquire the width information. This paper proposed a novel wrist pulse signal acquisition system, which could realize simultaneous measurements of the width and amplitude of dynamic pulse waves under different static forces. A tailor-packaged micro-electro-mechanical system (MEMS) sensor array was employed to collect pulse signals, a conditioning circuit was designed to process the signals, and a customized algorithm was developed to compute the width. Experiments were carried out to validate the accuracy of the sensor array and system effectiveness. The results showed the system could acquire not only the amplitude of pulse wave but also the width of it. The system provided more information about pulse waves, which could help doctors make the diagnosis.

## 1. Introduction

Pulse diagnosis, a non-invasive diagnostic method, is one of the most widely used diagnostic methods in traditional Chinese medicine (TCM), and its unique advantages are significant in the diagnosis of chronic disease. In pulse diagnosis, there are three pulse regions of the radial artery called Cun, Guan, and Chi [1]. TCM physicians put their fingers on the patient’s wrist and exert different static forces on the three regions. Usually, the range of static forces is divided into three segments, named Fu, Zhong, and Chen, which means the static force is light, medium, and heavy, separately. The strength of pulse wave varies with the static force. When the amplitude of the pulse signal sensed by the physician reaches its maximum, the static force is the best pulse-taking force, which corresponds to the depth of the radial artery. At this time, the patient’s pulse characteristics [2,3], including length, width, amplitude, speed, rhythm, intensity, etc., are captured and analyzed to help Chinese medicine practitioners diagnose the disease. These parameters are essential to mirror the condition of the cardiovascular system. For instance, the width is relative to blood pressure and blood flow [4].

However, in a sense, this type of diagnosis has a certain degree of artificial uncertainties and subjective error. In order to avoid the above cases and enable pulse diagnosis to serve health care better, digital pulse signal acquisition systems have been proposed and studied [5,6,7,8,9] in recent years. Furthermore, signal processing methods for pulse wave signals, such as machine learning, modified Gaussian models, and iterative sliding window algorithm, etc. [5,10,11,12,13,14,15,16,17], have also been extensively studied. Besides, the theory and applications of the harmonic analysis of wrist pulse waves have been proposed [18]. What’s more, in the researches [13,14,17,19,20,21], some links between specific diseases and pulse characteristics have been found. 

In TCM, the width of pulse waves isn’t precisely the diameter of the radial artery but the width of the radial artery and its peripheral tissues when the pulse beats. The width, which is tightly associated with blood pressure and blood flow, reflects the radial movement of the vessel and its surrounding tissues. Therefore, it is indispensable in the pulse signal acquisition system [4]. Varying with external static forces from the fingers, pulse waves contain not only the amplitude but also the width of it. For this reason, the acquisition system should be able to measure the width, the magnitude of pulse waves, and static forces synchronously.

With the development of sensors and manufacturing technology, more and more types of modern sensors, such as piezo-resistive [22,23,24], piezoelectric [25,26,27], photoelectric [28,29,30], and ultrasonic [31,32] sensors, have been applied to optimize the pulse wave acquisition systems. As a result, various parameters, including sensing accuracy, refresh frequency, and sensitivity, have been greatly improved. In recent years, many studies focused on sensor design in TCM, and pulse diagnostic devices have changed from single-probe type [4] and three-probe type [7,33] to five-probe type [6]. Among these studies, some researches [1,6] can measure the pulse waves under different static forces and compute their depth and frequency. However, most of them still cannot measure the width of pulse waves for all the probes are placed along the radial artery. For the purpose of obtaining the width of the pulse wave, it is necessary to develop a two-dimension sensor array with high sensitivity, wide range, and excellent reliability.

Besides, the sensor array in the pulse signal acquisition system should be able to simultaneously measure the static force applied by pressure device and the dynamic pulse wave under this force. According to our previous study, the static force is much higher than the pressure of the pulse wave [6]. Among the pressure transducers, strain gauges, piezoelectric sensors, capacitive sensors, and piezo-resistive sensors are most widely used. However, although strain gauge has been developed for many years, it still suffers a fatal defect that it cannot be made very small to form a dense sensor array. As for the piezoelectric sensor, it also has two serious drawbacks. Firstly, it cannot measure static force during the process of pulse signal acquisition because of losing charges quickly. Secondly, it can hardly keep consistency when it’s tiny, especially the polyvinylidene fluoride (PVDF) piezoelectric sensors.

Other optional sensors are capacitive sensors and piezoresistive sensors. Research on the capacitive sensor array has been carried out and received good results. The properties of the capacitive pressure transducer, such as sensitivity, repeatability, minimum element size, and temperature stability, are acceptable [9]. However, the pressure range is a little bit narrow, so additional pressure sensors are required to measure the static forces [16]. Besides, the structures of compound sensors like study [6,7] are involved; thus, it is easy to introduce interference from mechanical structures.

In order to solve the above problems, a three-dimensional wrist pulse signal acquisition system, based on a flexible micro-electro-mechanical system (MEMS) pressure sensor array, was proposed in this study. A conditioning circuit was designed for pulse signal processing. An analog-to-digital converter integrated into a microprocessor was utilized to convert the analog signal to a digital signal, which was then transmitted to a PC. An algorithm was developed to compute the width of the pulse wave and the amplitude of pulse wave. Experiments were carried out to validate the accuracy of sensors and system effectiveness. The results showed that the sensor array had good accuracy and repeatability and the proposed system could not only acquire the pulse waves under different static forces, meaning our proposed system could measure the depth, but also measure the width of the pulse wave.

## 2. System Design and Methods

### 2.1. System Overview

**Figure 1:**
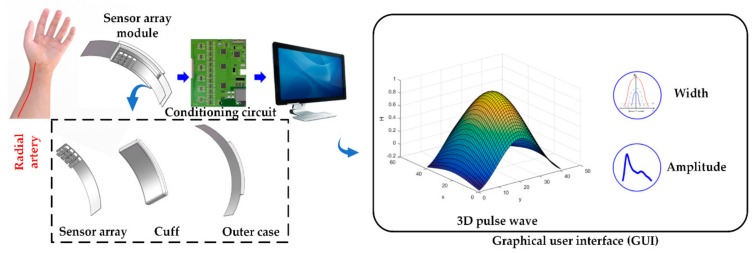
The proposed system includes a sensor array module, a conditioning circuit, and a GUI (graphical user interface).

The proposed acquisition system consisted of a sensor array module worn on the human wrist, a conditioning circuit, and a graphical user interface (GUI) on a terminal (e.g., PC or smartphone). The sensor array module is made of three parts: sensor array, which is used to collect pulse signal, a cuff to control the static force, and an outer case to fix the module. First, the pulse signal collected by the sensor array would be transmitted to the conditioning circuit and then transmitted to the terminal and eventually displayed on the GUI. The flow chart of the system proposed in this paper was divided into three parts: signal acquisition with the sensor array, signal processing with the conditioning circuit, and output display on the GUI. The proposed system is as shown in Figure 1.

The sensor array transduces the pressure into a voltage signal. The detected signal is then divided into static force and dynamic pulse wave through a lowpass filter, and then they are amplified separately. Finally, the signals are transmitted to the terminal and restored to a real-time 3D pulse wave, the width and amplitude under different static forces will be figured out and shown on the GUI right after. 

The sensor array is composed of 3 rows × 4 columns MEMS sensors. The row distance and column distance are 6.3 mm and 5.2 mm, respectively, where the distances are defined as the sensor center-to-center distance. The sensor array’s entire size is 18.6 mm × 20.3 mm, which is suitable to wear on the human wrist, and a single sensor’s size is 5.5 mm × 3.6 mm × 4.5 mm. The bending degree of the curved outer shell is tailored to fit the characteristics of most people’s wrists, making people feel comfortable when collecting the pulse signal, and this particular design can also maintain good contact between the sensors and human skin.

Although the range of human radial artery width in the wrist is approximately 1.3 mm–3.6 mm, which is less than the normal length of the sensor, we found that at least 3 of the 4 lateral sensors in a row could always detect the pulse wave under a certain static force in our preliminary experiments. Thus, we could perform the bicubic interpolation to create the 3D waveforms of pulse waves, ensuring that the sensor array is applicable for measuring the width of pulse waves.

### 2.2. Principle, Design, and Fabrication of Sensor Array Module

As the front end of the pulse signal acquisition system, the sensor is the critical component. Its multiple parameters determine the accuracy and repeatability of the MEMS sensor. The measurement range of the sensor should be more extensive than the variation range of static force, and the area of a single sensor should be small enough to obtain a high-density array. Besides, in our research, the package structure of the sensor should be tailor-made to ensure its good linearity, consistency, and stability.

**Figure 2:**
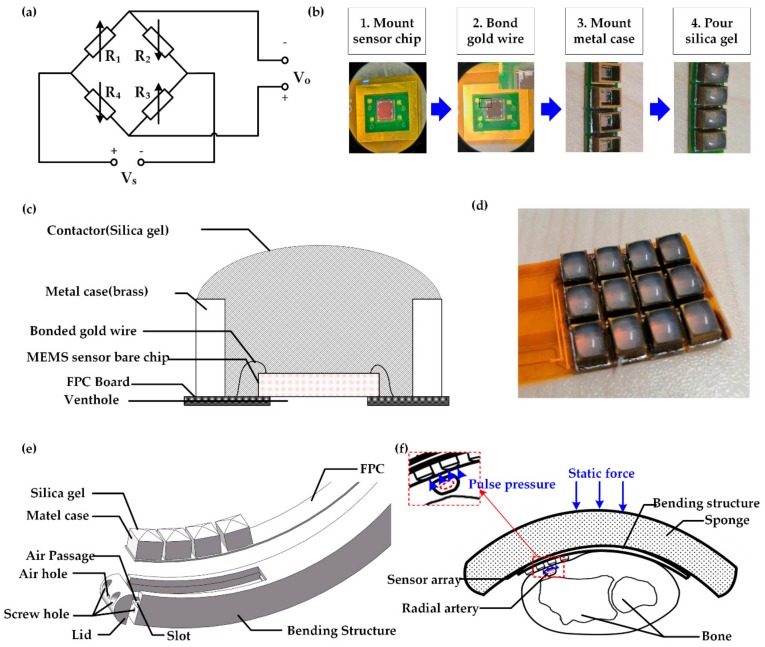
MEMS (micro-electro-mechanical system) sensor array. (**a**) Schematic diagram of the circuit structure of the sensor; (**b**) Fabrication process of sensor; (**c**) Section diagram of the package structure of sensor; (**d**) Photograph of MEMS sensor array; (**e**) Schematic diagram of bending structure; (**f**) Schematic diagram of the pressure device structure.

Based on the above analyses, a MEMS pressure sensor chip (MPS20N0100D, MEMStek Co., Ltd) was chosen as the sensitive element, which is widely used in non-corrosive, non-conductive atmospheric pressure environment and has good repeatability and long-term stability. As shown in Table 1, the parameters of the sensor chip met our requirements. The schematic diagram of the circuit structure of the sensor chip is shown in Figure 2a, where $V_{s}$ represents the voltage source, and $V_{o}$ means the differential output voltage. Four identical piezo resistors $R_{1}-R_{4}$ form a Wheatstone bridge. When values of four resistors vary with the external pressure applied to the sensor, the balance of the bridge will be broken, leading to changes in $V_{o}$.

In the design process, the key point is to find a suitable intermediate layer material to complete the pressure conduction of the wrist pulse. The physical and chemical characteristics of liquid silica gel, including transparency, resilience, yellowing resistance, thermal stability, water resistance, gas permeability, and heat aging resistance, have excellent performances; meanwhile, the viscosity is moderate and easy to handle. All these advantages indicate that it is a suitable material to conduct the pressure of the wrist pulse.

The complete processing flow is shown in Figure 2b. Firstly, to mount the sensor chip on the flexible printed circuit (FPC) board, the silver paste was applied on the welding pad of the FPC, with stiffeners made of glass-reinforced epoxy laminate material on the back of the pad. The chip-attached FPC board was then baked to solidify the silver paste. Secondly, the pins bonded on the chip to the corresponding pads of FPC with gold wire. However, the bonded gold wire could easily be broken when the FPC bends. In order to protect the sensor chip and the bonded gold wire, a metal case was mounted on their periphery in the third step. Finally, a proper amount of liquid silica gel was injected into the hole of the metal case using a needle tube, and then the FPC board was placed in the ventilation area, and there was a wait for about 3 to 4 h until the silica gel got solidified. Because the sensor is a gauge pressure sensor, one side of it was used to sense the pressure to be measured, and the other side needed to be exposed to air to sense atmospheric pressure through a venthole. Section diagram of the package structure of the sensor is shown in Figure 2c. The two-dimensional array, which consists of sensors of 4 rows and 3 columns, is shown in Figure 2d.

The performance of the sensor array depended not only on the sensor’s package structure but also on the design of the fixture and pressurizing device. In order to protect the sensor’s packing structure from damage, a bending structure was designed, as shown in Figure 2e. The slot was employed to accommodate the FPC welded with the sensors, and the lid was fixed on the bending structure (diameter: 80 mm, width: 9 mm, thickness: 5 mm) by a screw to seal the FPC. In addition, holes in the bending fabric and the lid were applied to allow the sensor to keep contacting air, making it work correctly.

Similar to the pressure device in our team’s previous study [4], an inflatable cuff was utilized to realize control of the static force. The structure of the pressure device is shown in Figure 2f. To acquire accurate pulse waves, a sponge sandwiched between cuff and bending structure evenly distributed the pressure onto the sensor array. Under the control of the software program, the static force exerted on the sponge reached a specific value and held for constant seconds to collect at least one entire period of pulse waves. The device provided static force ranging from 0 to 210 mmHg with a step of 35 mmHg during the whole collecting process, ensuring the variation curve of pulse waves under different static forces is completely obtained.

### 2.3. Circuit Architecture

**Figure 3:**
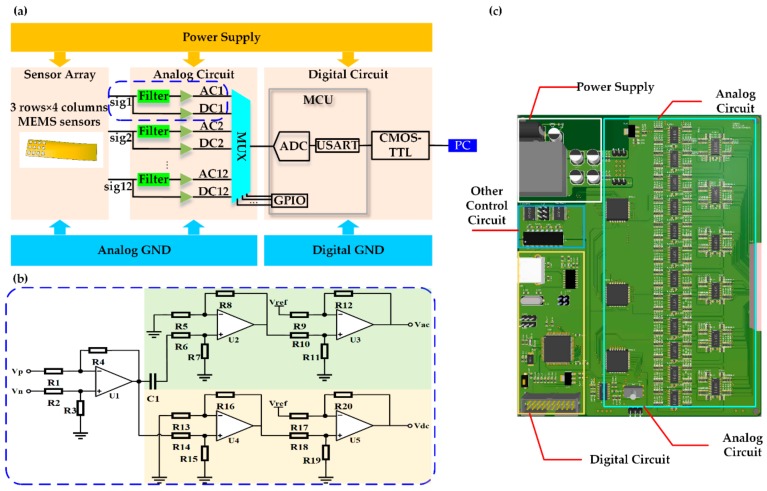
Schematic diagram of the circuit system. (**a**) Circuit system function diagram; (**b**) Detailed design in the blue dash frame in (**a**); (**c**) Printed circuit board layout view.

The circuit system was comprised of an analog circuit and a digital circuit; its schematic diagram is shown in Figure 3a. The design details of the circuit in the blue dash frame in Figure 3a is shown in Figure 3b. The voltage supplied to the sensor chip was 5 V; as a result, the sensor’s output signals Vn and Vp were both 2.5 V when the Wheatstone bridge was balanced. According to the preliminary test of the experiment, the pulse signal of the wrist radial artery in our acquisition system was less than 10 mV, which was much smaller than 2.5 V, so it was feasible to divide the signal into a dynamic pulse wave and the static force by a filter. An operational amplifier was employed to amplify the amplitude of weak pulse signals about 100 times to 0 V–3.3 V. Here, U1 was used to convert the differential signal that the sensor outputs into a single-ended signal, U2 was employed to amplify the dynamic pulse wave signal, and U3 was used to shift the amplified signal to the sampling range of analog-digital converter (ADC). Because the arm resistance of the sensor was 5 k ohm, R1 and R3 should be large enough to obtain the voltage signal produced by the sensor without distortion. The circuit composed of C1, R6, and R7 constituted a high-pass resistive-capacitive filter. Because the pulse signal’s frequency was between 0.8 Hz–2.5 Hz, the low cut-off frequency of the filter needed to be very low, and it was as small as 0.017 Hz in our design. 

Each array composed of 12 sensors outputted 12 differential signals, then each of them was filtered and amplified, generating an AC voltage signal (Vac ) and a DC voltage signal (Vdc). In the microcontroller unit (MCU), ADCs were too few to sample 24 channel signals—12 Vac signals and 12 Vdc signals—at the same time. Since only one ADC was used to collect 24 channel signals, the signals were converted into one signal by the multiplexer, and 24 channel signals were polled for sampling. Each time the polling was completed, a data frame formed by the 24 sampled signals would be eventually transmitted to the PC. The sampling frequency of the system was as high as 218 Hz, much higher than the pulse wave signal.

### 2.4. Algorithm for Calculating the Width of Pulse Wave

**Figure 4:**
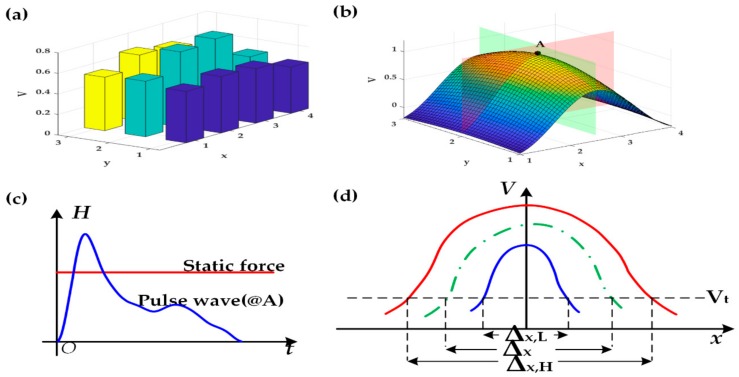
Algorithm for calculating the width of the pulse wave. (**a**) Signals received by the terminal; (**b**) Point A is the peak of 3D pulse wave; (**c**) Static force and Dynamic pulse wave at point A; (**d**) x-V cross-section view of the 3D pulse wave at point A (red in (**b**)).

Once the PC receives a data frame, the signals of 24 channels would be taken out in turn and stored in a data buffer; then, the signals would be restored to 12 static force signals and 12 dynamic pulse wave signals for further processing in the application program. In the application program, the collected signal is denoised, filters out the 50 Hz power frequency interference, removes baseline offset, and finally displays in the form of a 3D pulse wave. However, the width and amplitude of pulse wave could not be calculated directly for the x and y values of the 3D pulse wave and were discrete, as shown in Figure 4a. Bicubic interpolation was used to address this problem. First, the 50 Hz power frequency noise was filtered, then the cubic interpolation in the x-direction and y-direction was performed successively; finally, we got a smooth 3D pulse wave, as shown in Figure 4b. 

In order to calculate the parameters of the pulse wave, according to [4], we first located the peak point of the 3D pulse wave, which had the most significant change in the 3D pulse wave graph and marked it as point A. The diagram of the static force signal and the dynamic pulse wave signal at point A is shown in Figure 4c. When the amplitude of pulse wave at point A reached its peak, the cross-sections parallel to x-V, shown in Figure 4d, were used to calculate the width. The width of the pulse wave was determined by a specific threshold called $V_{t}$, which was vital to extract and calculate physiological parameters. In Figure 4d, the green dot-dash line represents the cross-section of a 3D pulse wave, whereas the solid red line and the solid blue line mean the upper and lower thresholds of the pulse wave width of healthy people, respectively, so the width of the pulse wave collected from healthy people should be within the range of [$\Delta_{x,L}$, $\Delta_{x,H}$]. The width is calculated by $W=\Delta_{x}\times D_{x}$, where $D_{x}$ is the column distance 5.2 mm.

## 3. Experimental Results and Discussion

### 3.1. Calibration of Packaged Sensor

In order to test the performance of the sensor, a piece of calibration equipment was employed to test the sensor repeatedly under certain standard conditions. The output voltage of the packaged sensor (marked as V) varied with the exerted pressure (marked as F). If the sensor was linear, V and F satisfied V=a∗F, where a represents the sensitivity of the sensor. In case of very slight or strong pressure, the linearity was used to disappearing in real conditions.

**Figure 5:**
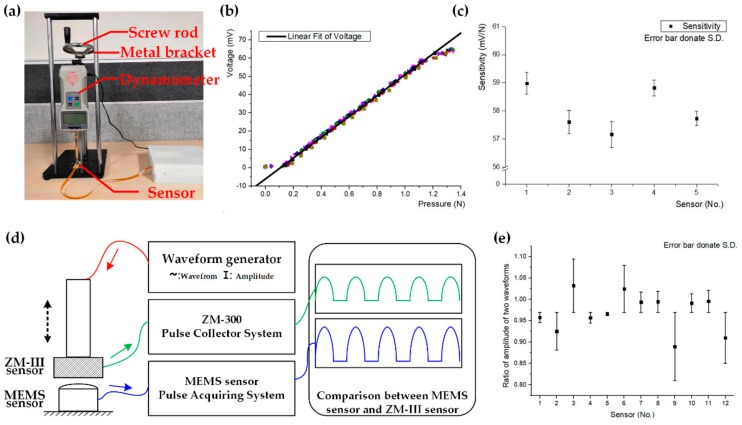
Calibration of the sensor. (**a**) The device in the calibration experiment of sensors; (**b**) Linear fit of Voltage-Pressure; (**c**) Sensitivity of each sensor; (**d**) Comparison experiment of dynamic characteristics between MEMS sensor and ZM-III sensor; (**e**) Result of comparison experiment.

The calibration device is shown in Figure 5a. In the calibration experiment, we adjusted the screw rod manually on a designed metal bracket, and the slider on the guide rail moved up and down. When the dynamometer fixed on the slider contacts the sensor, the interaction between the dynamometer and the sensor could be measured directly. Six sensors were randomly selected for testing, and each sensor was tested 10 times.

The least-square method was used to fit the test data in the forward and reverse stroke linearly, then we could obtain the slope of the regression line, which was the sensitivity of each sensor, as shown in Figure 5b,c. Figure 5b shows the test data of 10 times for one sensor, and Figure 5c shows the sensitivity of each sensor. The diameter of the contact surface between the MEMS sensor and the sensor of the dynamometer was about 4 mm, and the effective range of pressure was about 0.1–1.15 N, which is the linear area in Figure 5b, so the range of pressure that the sensor could detect was about 7.96–91.51 KPa. The average sensitivity of the six sensors was about 58.06 mV/N, and the standard deviation of the sensitivity of the six sensors was 0.71 mV/N. 

According to the above results, the maximum pressure that the sensor could bear was 91.51 kPa, which covered the pressure range of TCM pulse diagnosis. The outstanding linearity of the sensor accurately reflected the linear relationship between the pressure and the output voltage signal, which satisfied our demand for pulse signal acquisition. The small difference in sensitivities of six sensors was only about 2.2%, indicating that sensors had a high consistency.

The repeatability of the sensor indicated the difference in the performance of the sensor in many experiments, which also revealed the stability and reliability of the sensor. Equations for calculating sensor repeatability are shown as follows:

(1) $$s_{ui}^{2}=\frac{1}{m-1}\overset{m}{\underset{j=1}{\sum }}{\left(y_{uij}-{\bar{y}}_{ui}\right)}^{2}$$

(2) $$s_{di}^{2}=\frac{1}{m-1}\overset{m}{\underset{j=1}{\sum }}{\left(y_{dij}-{\bar{y}}_{di}\right)}^{2}$$

(3) $$s\;=\sqrt{\frac{1}{2n}\overset{n}{\underset{i=1}{\sum }}\left(s_{ui}^{2}+s_{di}^{2}\right)}$$

where y is the measured voltage, $s_{ui}$ and $s_{di}$ are the variances of forward and reverse travel of the measurement point i, respectively, m represents the number of repetitions of test experiment, in this experiment, the value is eight, n is the number of measurement points. 

To calculate the sensor repeatability, like the method used in the above experiments, we selected six sensors randomly, each of which was tested eight times. Using Equations (1)–(3), we obtained the repeatability values of the six sensors, which were 6.4%, 4.1%, 3.4%, 4.7%, 4.2%, 4.8% respectively. The average repeatability was 4.6%, which reflected the repeatability was good.

In order to further validate the dynamic characteristics of the sensor, we compared the performances of our single sensor with the commercial sensor of ZM-300 [34,35], which meets technical standards for the pulse detector of traditional Chinese medicine and is commonly considered as a standard pulse signal detection system in TCM. A comparison experiment was carried out, as shown in Figure 5d, a waveform generator was used to generate a specific wave, which led to the up-down movement of ZM-300 to simulate human pulse wave, and then the interaction between ZM-300 and our sensor, which increased as the amplitude of the waveform generator’s output increased, was measured by the two sensors simultaneously, ensuring that the pressures the two sensors detected are always same. The default unit of ZM-300’s output, which is $g/cm^{2}$, was transformed into kPa to keep consistent with the output of our MEMS sensor. Then, the ratio of the amplitude of the waveforms captured by the two sensors was calculated. We measured 12 sensors, and each sensor was tested six times. 

The amplitude of the interaction between the two sensors was within the range 11.9 kPa, which was much larger than the magnitude of the general pulse wave signal (~1 kPa), ensuring that the pulse wave could always be recorded losslessly. The 12 sensors’ test results are shown in Figure 5e. The mean ratio of 12 sensors was 0.97, indicating that the MEMS sensor’s linearity differed little from that of ZM-300, and it was applicable for collecting dynamic waves. The standard deviation of 12 sensors was 4.36%, which proved that the consistency of sensors was excellent.

### 3.2. Static Force and Dynamic Pulse Wave Measurement

**Figure 6:**
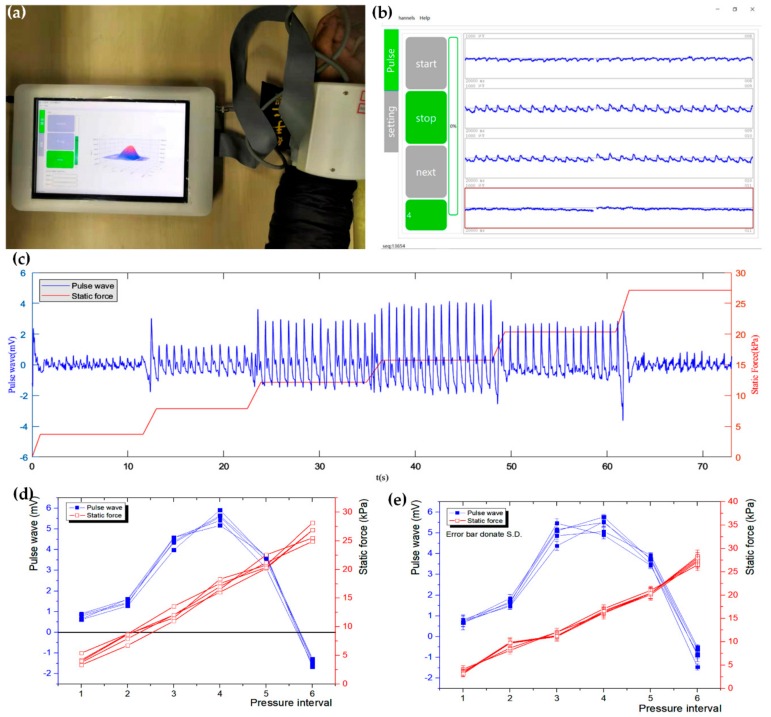
Static force and dynamic pulse wave measurement. (**a**) Photograph of our system, which shows the real-time 3D pulse wave; (**b**) A screenshot of the 2D pulse wave on the GUI, which shows three of four lateral sensor in a row in actual situation; (**c**) Pulse waves under different static forces during wrist pulse signal collecting; (**d**) Dynamic pulse wave and static force of a volunteer’s five tests; (**e**) Dynamic pulse wave and static force of six volunteers.

To validate the system’s effectiveness, we recruited six volunteers into the experiments. The sensor array attached to an inflatable cuff was tied to the volunteers’ wrists. The cuff pressure exerted to the volunteers’ wrists was gradually increased at a step of 35 mmHg, each time stopping for a few seconds to obtain steady pulse wave signals until the pressure value was 210 mmHg. A photograph of our system, which showed the real-time 3D pulse wave, and a screenshot of the 2D pulse wave on the GUI, which showed three of four lateral sensors in a row in the actual situation, are shown in Figure 6a,b.

Because there was a sponge between cuff and wrist, the pressure of the cuff was not equal to the static force applied to the wrist, so the pressure of the cuff was unable to represent the static force. However, the static force could be obtained directly by extracting the data from the conditioning circuit. As for dynamic pulse wave, firstly, the noise from the power supply was filtered by low pass filter with high cut-off frequency of 40 Hz, and then the baseline, which means zero drifts of sensors, was removed by high pass filter with low cut-off frequency of 0.2 Hz, and the available dynamic pulse signal was obtained.

Like the above algorithm for calculating the width of the pulse wave, we performed bicubic interpolation and obtained the 3D waveform. Then, we located the peak point A of the 3D pulse wave. Pulse waves under different static forces at A are shown as Figure 6c, showing the trend of pulse wave while the static force is increasing. When the static force gradually increases from zero, the amplitude of the pulse wave rises until it reaches its maximum, and after that, the magnitude of the pulse wave would decrease progressively, which reflects the static force inhibits the pulse beat. The amplitude goes negative then, which means the pulse beat gets weaker than the interference, which is caused by the physical connection with the pressure device. The results showed that the packaged sensor was capable of collecting static force and dynamic pulse waves synchronously.

To estimate the repeatability, we recruited six volunteers, and each of them was tested 5 times. The amplitude of pulse wave and static force of a volunteer’s five tests are shown as Figure 6d. The standard deviations of six pressure intervals were 15.4%, 8.5%, 5%, 4.5%, 6.3%, and 9.7% respectively. When the force exerted on the wrist was very small, the interference brought from noises caused by the volunteer’s slight movement was relatively larger, leading to a higher deviation. Though it’s only a little high, yet we actually paid more attention to the pulse waves with high amplitude because they are interfered with by fewer noises but more meaningful. The dynamic pulse wave and static force of six volunteers are shown in Figure 6e. The largest standard deviation of the pulse wave and static force under 2–6 pressure intervals was 9.7%, indicating that the repeatability of the system was good. In summary, the sensor array could measure pulse waves under a wide range of static forces.

### 3.3. Amplitude and Width Measurement of Pulse Wave

**Figure 7:**
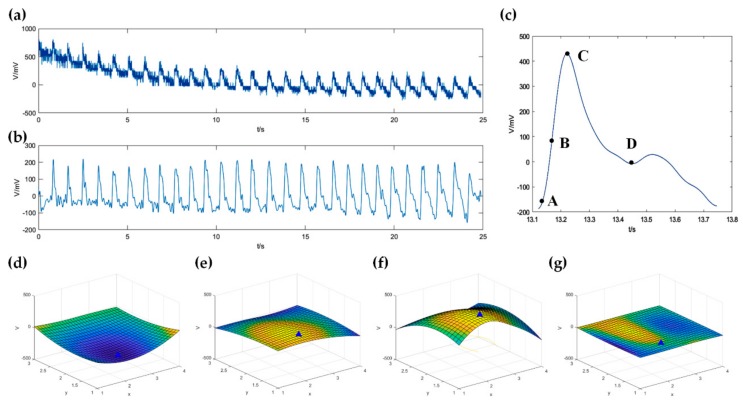
The preprocessing algorithm of the pulse wave signal. (**a**) Original signal; (**b**) Signal filtered by low-pass filter and high-pass filter; (**c**) A period of pulse wave after bicubic interpolation; (**d–g**) are 3D waveforms at four different times A, B, C, and D in a period of the volunteer’s pulse wave in (**c**).

In our algorithm, the signals of 12 channels were filtered out the 50 Hz power frequency interference by a low-pass filter (cut-off frequency equals to 40 Hz), then the baseline offset was removed by a high-pass filter (cut-off frequency equals to 0.2 Hz). One channel’s original signal and the signal after the high-pass filter is shown as Figure 7a,b, separately. After bicubic interpolation, a period of pulse wave signal is shown as Figure 7c. At four different times A, B, C, and D, the corresponding 3D waveforms are shown as Figure 7d–g. The blue triangle in the figures represents the peak point, which has the greatest beating amplitude in the 3D pulse wave. The amplitude of the pulse wave could be calculated by the peak-to-peak value, which was about 536.2 mV, as shown in Figure 7c.

**Figure 8:**
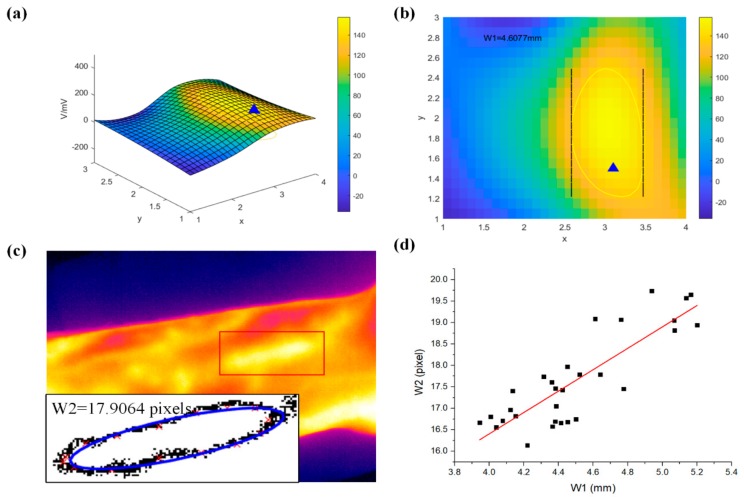
The width measurement of the pulse wave. (**a**) 3D waveforms of the pulse wave. (**b**) the projection view of (**a**) and the width of pulse wave (W1). (**c**) the corresponding infrared image and the width of pulse wave (W2). (**d**) The linear relationship between W2 and W1.

To validate the effectiveness of the measured width, we recruited 10 volunteers and measured both their pulse waves and the infrared image of their wrists three times, respectively. First, according to the above algorithm, we obtained the 3D waveforms shown in Figure 8a, and the corresponding projection view is shown in Figure 8b. Then the width, marked as W1, was calculated by the yellow contour, which is drawn at the threshold. As for the infrared image shown in Figure 8c, we took three steps to obtain the width of pulse wave: first, the area of the pulse wave was manually located; second, after graying the image, we used the threshold to determine the boundary of the area; finally, we used the elliptic equation to fit it and got the length of the short axis, which is the width of the pulse wave, marked as W2. The scatter graph of W1 and W2 is shown as Figure 8d, then we fitted the data linearly, and the square of R we obtained was 0.75.

In fact, because the resolution of the infrared camera is limited, the measurement error of the width detected using the infrared image was a little large, which resulted in a little low correlation coefficient calculated above. Despite this, it was still higher than seven, indicating that the relation between these two variables was interrelated positively, proving that our measured width was sufficient.

**Figure 9:**
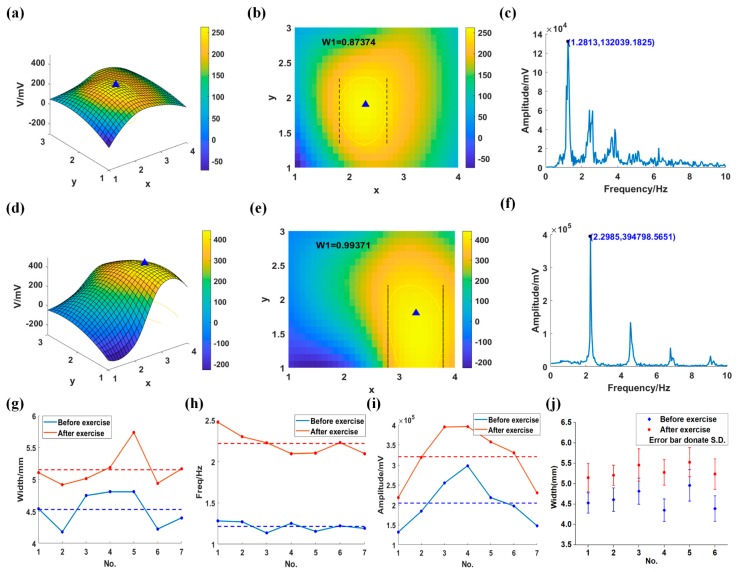
The difference of pulse wave between sitting for 20 min and running for 10 min. (**a**,**b**) are pulse wave of sitting for 20 min; (**d–f**) are pulse wave of running for 10 min; (**a,d**) are the 3D waveforms; (**b,e**) are the projection view of (**a**) and (**d**); separately, (**c,f**) are the frequency spectrums of pulse wave; (**g–i**) are the width, frequency, and amplitude of the pulse waves; (**j**) is the width of six volunteers.

To further test the clinical significance, we measured pulse waves of six volunteers before and after exercise, and each of them was tested for seven times. The results of a volunteer are shown in Figure 9. First, we obtained the 3D waveforms of before and after running for 10 min, shown as Figure 9a,d, respectively. The corresponding projection views are shown in Figure 9b,e. The area of the yellow circle determined by the threshold and the amplitude of pulse wave after exercise were more extensive than those before exercise. After fast Fourier transform (FFT), we got the frequency spectrum of the pulse wave before and after exercise, as shown in Figure 9c,f. In the spectrum diagram, we calculated the amplitude and the signal frequency. 

Compared to the data before exercise, the amplitude and the frequency were much larger. In our experiment, the width, frequency, and amplitude of a volunteer’s seven tests are shown in Figure 9g–i, respectively. Width, frequency, and amplitude were obviously different between before and after exercise, indicating that the system could distinguish pulse waves with different amplitude, width, and frequency. The standard deviations of the width before and after running were 5.5% and 4.9%. The average values of the width of six volunteers before and after running are shown in Figure 9j, and the maximum standard deviation was 7.8%, indicating that the repeatability was good.

In summary, Table 2 lists the apparatus that have been reported in collecting pulse wave of the radial artery. Compared with other pulse signal acquisition systems, the proposed system had several advantages. Firstly, compared with the device proposed in the study [16], the pulse wave and the static force from the pressure device could be simultaneously collected by a MEMS pressure sensor array, which has a wide range, high sensitivity, and excellent repeatability. What’s more, its quite mature technology ensured its good consistency. The designed system had collected the pulse signal of volunteers under different static forces, showing the trend of pulse waves under various static forces entirely and accurately, so this study had higher practical values to TCM diagnosis.

Secondly, pressure sensors had excellent stability for the unique protective parts and specially designed bending structure. Pressure sensors were mounted on an FPC, so the sensor array could be bent to fit tightly into the human wrist, ensuring the signal intensity [36]. Furthermore, the MEMS pressure sensor could collect repeatable pulse data accurately.

Thirdly, compared with our previous study [6], the width and amplitude of the wrist pulse wave could be measured by the sensor array, which formed a three-dimensional wrist pulse wave, providing abundant information of pulse wave. More pulse information helps the doctor make a more accurate diagnosis. Also, compared with the traditional pulse waveform, the visual perception of the three-dimensional pulse wave is more intuitive and convincing.

## 4. Conclusions

This study aimed to realize a three-dimensional wrist pulse signal acquisition system, which could collect static force and the width and amplitude of dynamic pulse waves simultaneously. The package structure of the sensor was designed to ensure its high sensitivity, excellent consistency, and exceptional repeatability; as a result, the sensor array could collect the pulse wave under a wide range of static force accurately. In addition, the variation trend of wrist radial artery pulse waves under different static forces showed that the system was in accordance with the pulse acquisition process of TCM, and was conducive to further exploring the relationship between pulse signal and health condition. Finally, a three-dimensional pulse map could not only visually and concretely show the pulse wave of the wrist radial artery but also provide the width and amplitude of pulse wave, providing a novel reliable auxiliary approach for the diagnosis of TCM doctors.

In the future, we plan to extract more valuable information from the data, including not only the parameters of time domain but also the features of frequency domain since the study [16] have reported that the heart itself and the matching condition of the heart with the arterial system are mutually influenced to generate the harmonic spectrum of pulse wave. Moreover, we would apply machine learning and deep learning algorithms in the classification of pulse waves, which could be helpful in finding the connections between cardiovascular disease and pulse waves.

## 5. Patents

The works presented in this paper are subject to pending China and international patents filed by Institute of Microelectronics of Chinese Academy of Sciences (IMECAS) in China (201910570914.4, 201910570913.X, PCT/CN2019/093154, and PCT/CN2019/093156).

## Figures and Tables

**Table 1:** Parameters of MEMS (micro-electro-mechanical system) sensor.

Variables	Value
Full-scale range	100 KPa
Resistance	5 kΩ
Full scale output	80 mV
Nonlinearity	0.3 %FS
Stability	MAX: 0.2 %FS/Y
Hysteresis	MAX: 0.2 %FS/Y
Work temperature	–40−125 °C
Pressure resolution	0.025 KPa

**Table 2:** The performance comparison of acquisition systems.

Researcher	Number of Length Sensing Elements in an Array	Number of Width Sensing Elements in an Array	Needs Extra Sensor(s) to Detect Static Force	3D Pulse Wave
Liu Su et al. [6]	5	0	Yes	No
Peng Wang et al. [22]	0	12	Yes	No
Chung-Shing Hu et al. [9]	4	3	Yes	Yes
Our proposed	3	4	No	Yes
